# Supplementary material for: Diverse monogenic subforms of human spermatogenic failure
Source: Nat Commun. 2022 Dec 26;13:7953. doi: 10.1038/s41467-022-35661-z (PMC9792524; doi:10.1038/s41467-022-35661-z)
Supplement: Supplementary file 1 — Supplementary Information [file 41467_2022_35661_MOESM1_ESM.pdf]

SUPPLEMENTARY INFORMATION FOR:

Diverse Monogenic Subforms of Human Spermatogenic Failure

This PDF file includes:

1. Supplementary Figures 1 to 8
2. Supplementary Tables 1 to 7
3. Supplementary Discussion
4. Supplementary References

SUPPLEMENTARY FIGURES

a

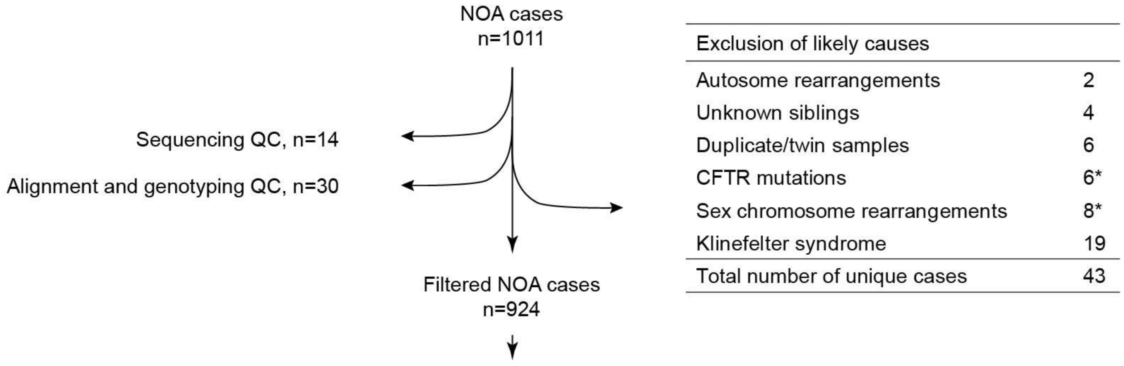

b

Country	No. of centers	Recruitment center	No. of cases
Australia	1	Monash University, Clayton, Victoria, Australia	88
Canada	1	Mount Sinai Hospital, University of Toronto, Toronto, ON, Canada	35
Denmark	1	Copenhagen University Hospital - Rigshospitalet, Copenhagen, Denmark	87
Estonia	1	Tartu University Hospital, Tartu, Estonia	82
Portugal	2	Faculdade de Medicina da Universidade do Porto, Porto, Portugal	147
		Instituto Nacional de Saúde Dr Ricardo Jorge, Lisboa, Portugal	128
Spain	1	Instituto de Investigaciones Biomédicas Sant Pau (IIB-Sant Pau), Barcelona, Catalonia, Spain	46
USA	4	Stanford University School of Medicine, Stanford, CA, USA	10
		University of Utah School of Medicine, Salt Lake City, UT, USA	62
		Washington University, St. Louis, MO, USA	34
		Weill Cornell Medicine, New York, NY, USA	205

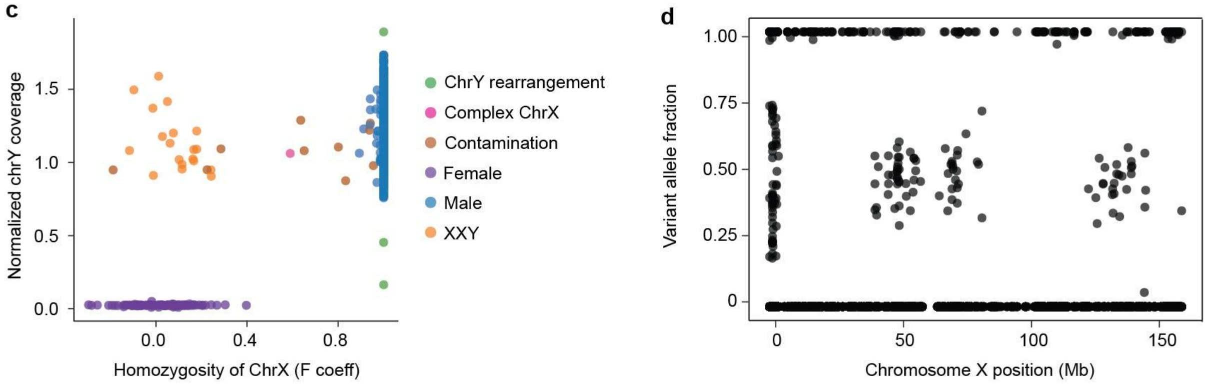

Supplementary Fig. 1. Filtering procedures of the GEMINI study cohort and screening for known genetic causes based on WES genotype call data. a, Sample filtering steps based on the WES data. Two cases harbored a combination of sex chromosome aberrations and a deleterious *CFTR* variant (asterisk; see Supplementary Discussion). **b**, Distribution of studied NOA cases (n=924) across the participating centers of the GEMINI study. **c**, Klinefelter syndrome (47,XXY karyotype) and large rearrangements on sex chromosomes were detected based on i) the CNV data and ii) the ratio of chromosome Y coverage (normalized to autosomes) and inbreeding coefficient F of chromosome X. Klinefelter syndrome was called if chrX $F<0.5$ and chrY coverage >0.6 and compared to the clustering observed for normal karyotypes of NOA

cases (chrX *F*>0.5 and chrY coverage >0.6) and for women analyzed on an identical sequencing platform (n=96; chrX *F*<0.5 and chrY coverage <0.6). A NOA case with suspected large X chromosome rearrangements (‘Complex ChrX’) was identified as an outlier with respect to the inbreeding coefficient. Samples affected by DNA contamination (confirmed by FREEMIX tool, Methods) were not considered as candidates for sex chromosome aberrations. **d**, Distribution of variant allele fraction of all variants on the X-chromosome in the carrier of complex rearrangements.

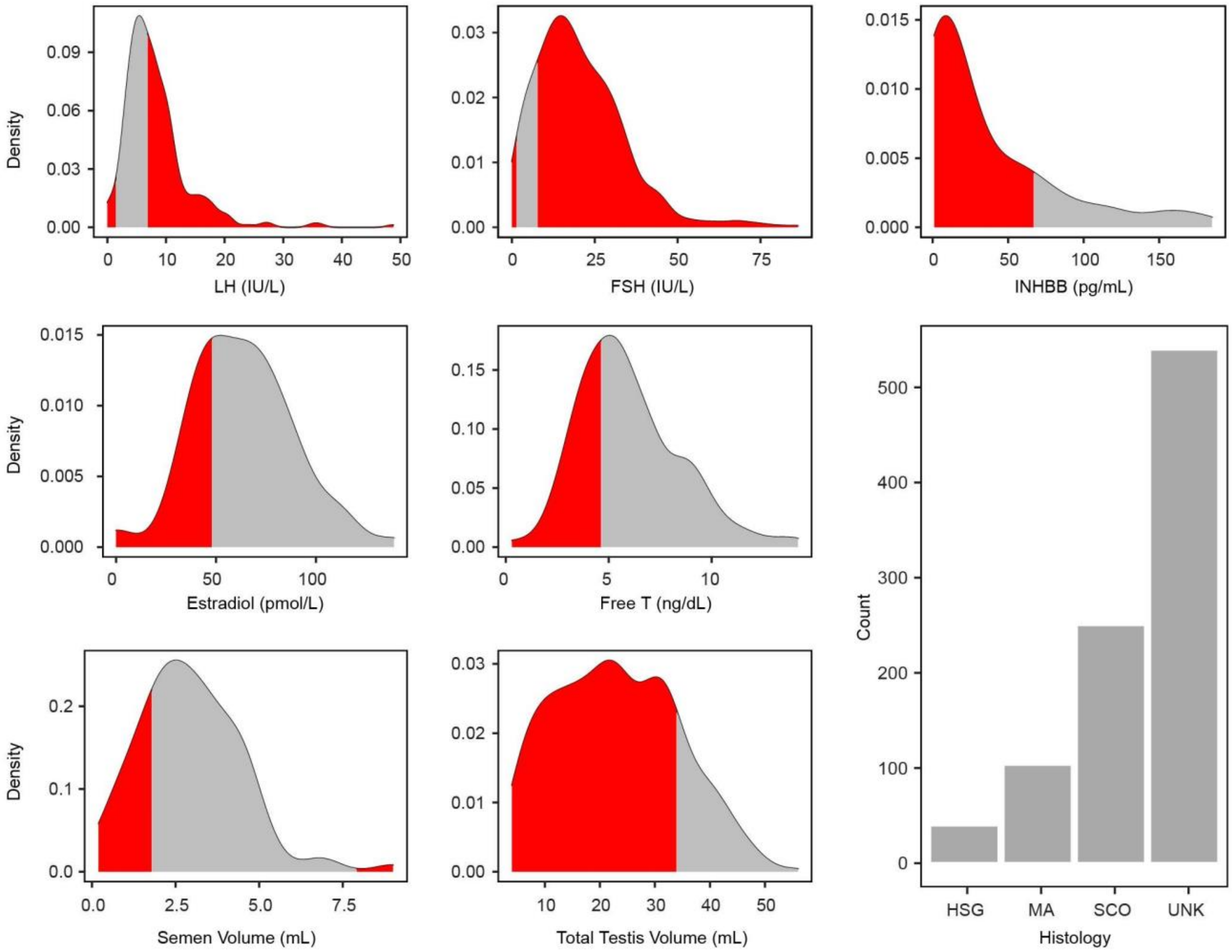

Supplementary Fig. 2. **The distributions of reproductive hormone measurements, ejaculate volume and total testis size of 924 men included in the study.** Measurements that fall outside of the published normal ranges (Supplementary Table 1) are shaded in red. HSG, hypospermatogenesis; MA, maturation arrest; SCO, Sertoli cell only; UNK, unknown testicular phenotype.

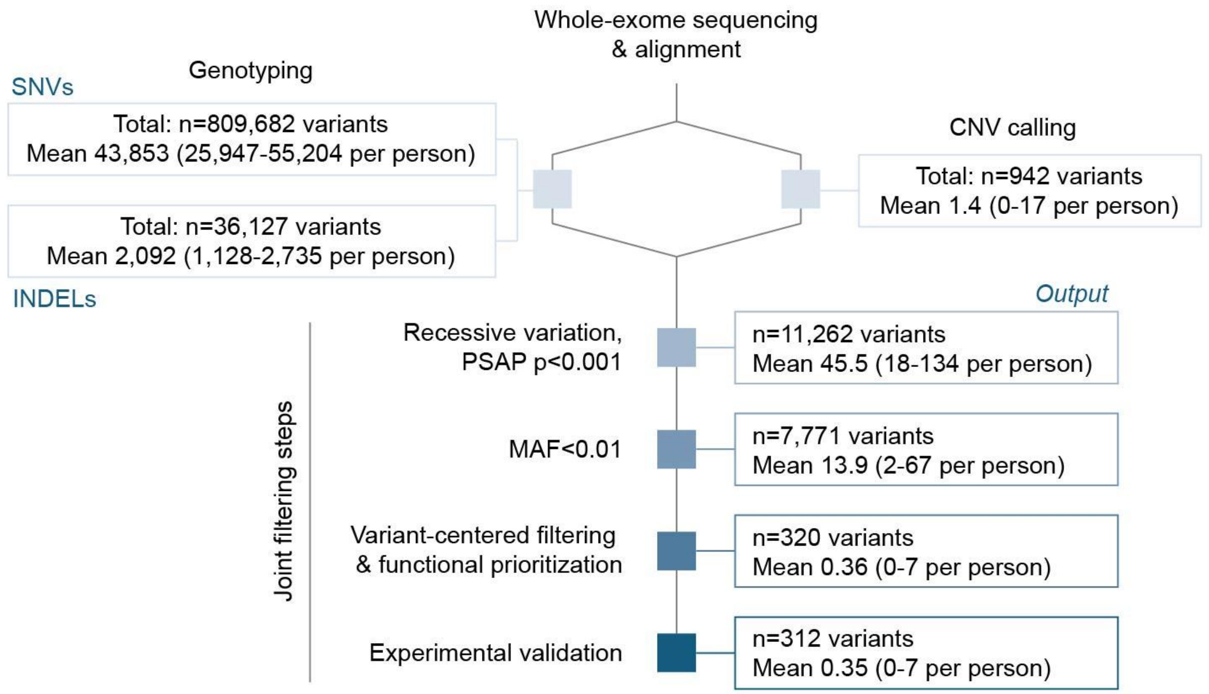

Supplementary Fig. 3. **Detection and filtering of variants in 924 NOA cases included in the study.** Whole-exome sequencing alignment was followed by detection and quality filtering of SNVs/INDELs and CNVs independently and subsequently combined for downstream filtering steps. PSAP was used to prioritize likely pathogenic genotypes, followed by selection of rare variation and exclusion of likely false positives (see details in Methods). The functional prioritization aimed to aggregate variation most likely to disrupt spermatogenesis. Total of 40% of all prioritized genotypes, predominantly LoFs and recurrent or poorly covered positions (read depth<20), were subjected to experimental validation by Sanger sequencing (SNVs and INDELs) or +/- PCR amplification (CNVs; Methods).

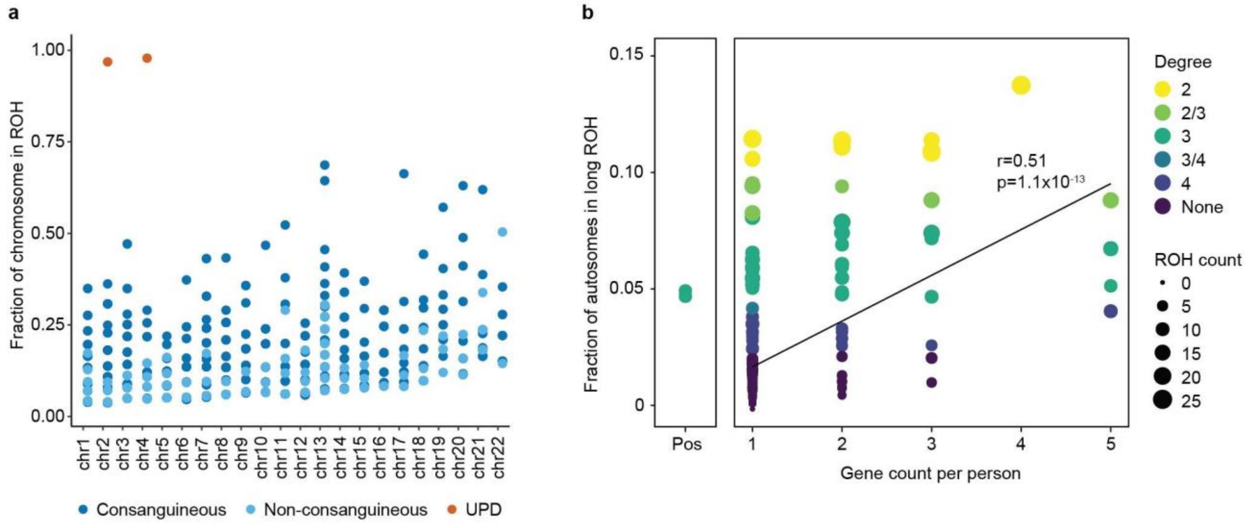

Supplementary Fig. 4. **Uniparental isodisomy (UPD) and consanguinity in NOA cases. a**, The fraction of chromosomes found in long runs of homozygosity (ROH) highlight two cases with UPD of chromosomes 2 and 4. **b**, Multiple disease genes are more likely to be identified in patients with multiple long runs of homozygosity (ROH) (Pearson's R=0.51, $P=1.1x10^{-13}$). Degree, estimated degree of parental relationship; Pos, siblings with known 3rd degree of consanguinity.

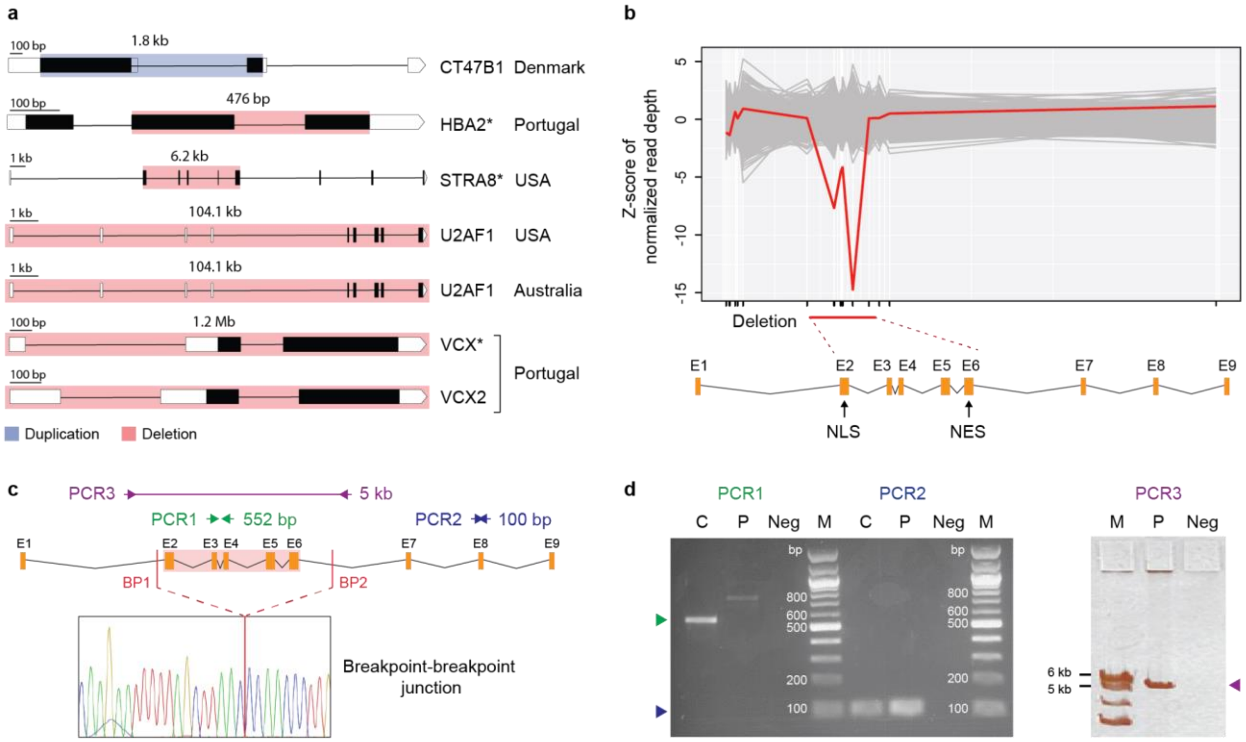

Supplementary Fig. 5. Prioritized copy number variants in NOA cases and validation of *STRA8* deletion. a, CNV events involving prioritized NOA genes. *HBA2* gene is exclusively expressed in the whole blood (GTEx database) and likely represents a false positive finding. *Validated by PCR. **b**, Z-scores of normalized read depth of exome sequencing data around the *STRA8* locus, plotted against the *STRA8* gene model. NLS; nuclear localization; NES, nuclear export signal. **c**, Schematic representation of experimentally mapping the *STRA8* deletion in the carrier DNA. Three PCR reactions were used with primers anchoring in exons E3-E4 (PCR1, inside the predicted deletion region (pink box) spanning chr7:134,925,271-134,931,454, hg19) expected to be amplified in control (C) only and within exon E8 (PCR2, outside the deletion) amplified in both control and the patient. Third PCR reaction (PCR3) was designed to span the deletion region and enabled to determine the breakpoint-breakpoint junction of the deletion by Sanger sequencing. The deletion breakpoints were mapped at positions chr7:134925172 (BP1, 98 bp from E2) and chr7:134933449 (BP2, 2050 bp from E6). **d**, As expected, PCR1 did not yield a PCR product in the patient (P; the observed 800 bp band is a non-specific product), whereas PCR2 reaction was amplified in the patient. The PCR3 reaction spanning the deletion breakpoints resulted in a 5200 bp fragment in the patient when the expected size in control (C) was 13 kb. Experiment repeated twice with the same result. Neg, no DNA control; M, marker.

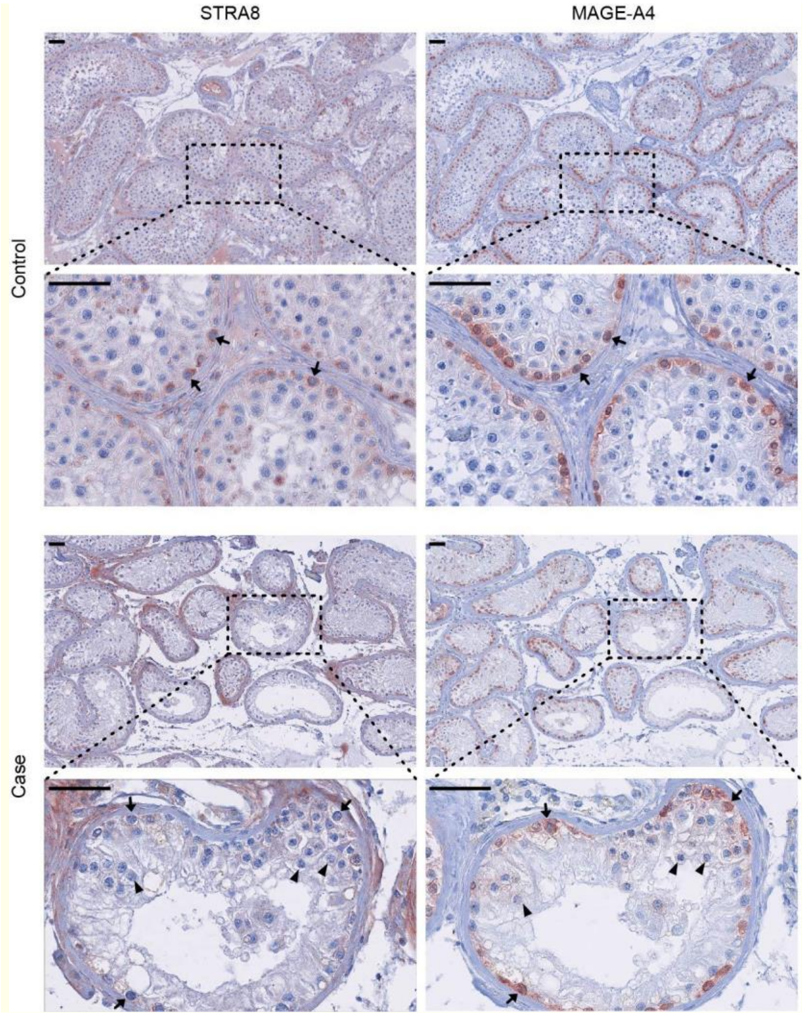

Supplementary Fig. 6. **STRA8 and MAGE-A4 staining of testicular tissue.** The carrier of *STRA8* deletion displayed significantly reduced staining of STRA8 in spermatogonia (arrows) compared to the control with normal spermatogenesis. The *STRA8* deletion caused a maturation arrest phenotype, whereby the tubules predominantly contained spermatogonia, stained with spermatogonia-specific marker MAGE-A4, and occasional pre-pachytene spermatocytes (arrowheads). Three different sections of the controls (n=8) and the case (n=1) showed consistent staining patterns. Non-specific background staining is observed particularly in peritubular and Leydig cells in spite of extensive optimization of the only commercially available antibody. Scale bars represents 50μm

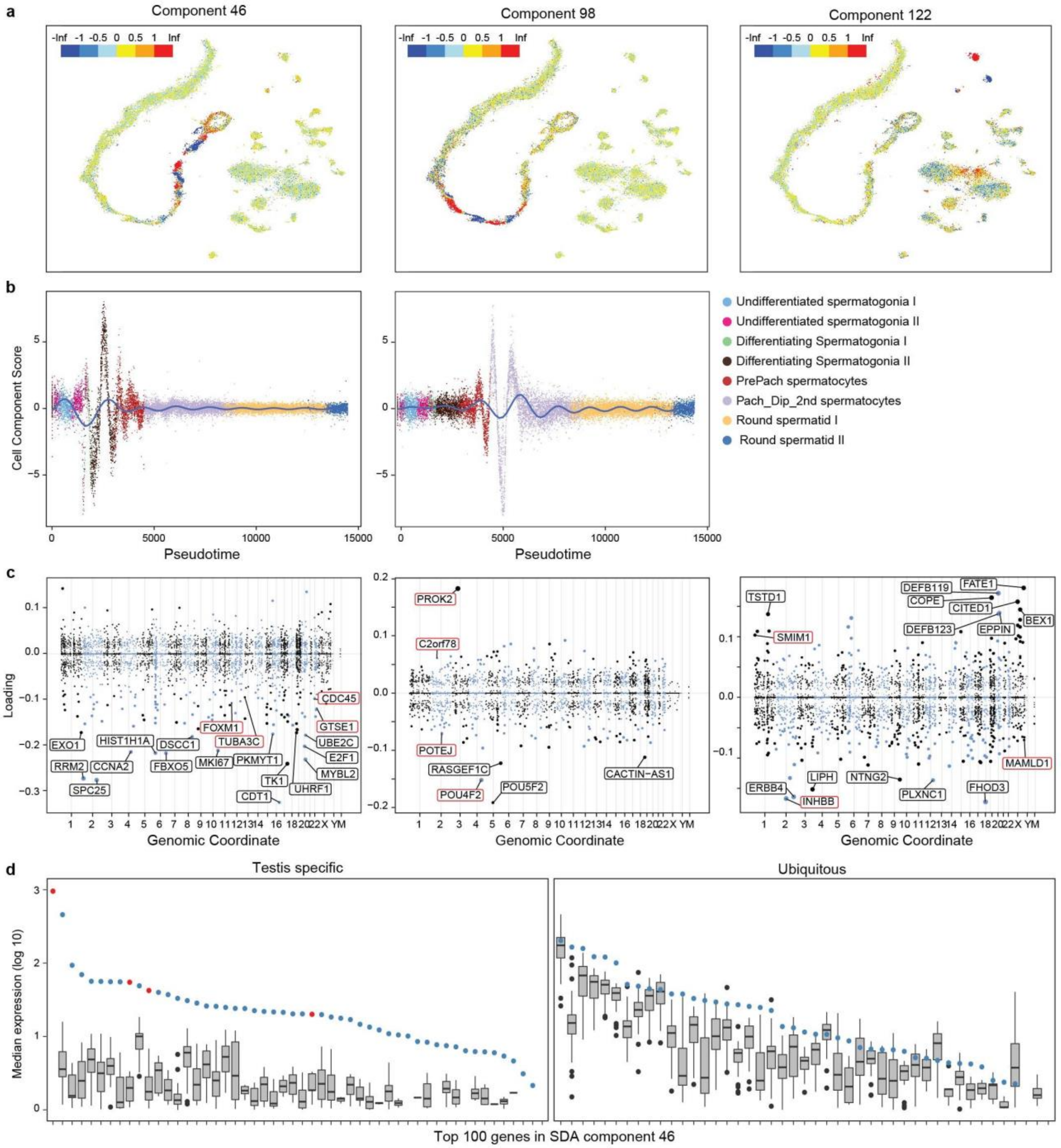

Supplementary Fig. 7. Examples of three potential subforms of azoospermia corresponding to defects in type B spermatogonia, spermatocytes, or Sertoli cells. a, Projection of cell loadings for the SDA components 46, 98 and 122 plotted in a t-SNE space. SDA component 46 appears to encode a set of genes specifically expressed in differentiating spermatogonia, whereas component 98 involves spermatocytes and component 122 involves Sertoli cells. tSNE plots use the same coordinates as in Figure 3 of the main text. **b**, Pseudotime (or germ cell development

trajectory) plots for components 46 and 98. Note the primary cell type(s) expressing component 46 in a cycling manner, which probably correspond to the two mitotic divisions of Type B spermatogonia prior to meiosis. Like component 98 shows two cycles that may correspond to meiotic divisions. **c**, Manhattan plots showing the genes in the SDA components 46, 98 and 122, which can be used to infer the biological function(s) encoded by a component. Gene loadings corresponding to genes prioritized in the NOA cases are highlighted in red. **d**, Genes loading on component 46 can be divided into two groups; those with testis-enriched expression and those that are ubiquitous across the body. Each column corresponds to the expression pattern of a single component 46 gene: the box-and-whiskers plot summarizes the median gene expressions across 52 tissues extracted from the GTEx RNA-seq dataset (Methods)[1], while each point corresponds to the expression of that gene in testis alone. Red points correspond to genes identified as associated with male infertility in this study. Only expression levels above 1 transcripts per million (TPM) are visualized. The center of the boxes indicate the median. lower and upper hinges of the boxes correspond to the 25th and 75th percentiles, the whiskers extend from the hinge to the largest (upper whisker) or lowest (lower whisker) value no further than 1.5x inter-quartile range from the hinge.

a

Gene; Transcript ID	Variant*	gnomADv2.1.1 popmax MAF	Inheritance mode	Degree consanguinity	Descent†	Testicular phenotype	Cohort
PLD6, ENST00000321560.3	p.[(L98R)];[(V118M)]	$3.1x10^{-4}$; novel	Chet‡	-	Hispanic	n/a	USA
	p.[(L98R)];[(P58fs)]	$3.1x10^{-4}$; $3.1x10^{-5}$	Chet‡	-	Caucasian	n/a	USA
PNLDC1, ENST00000610273.1	**p.L34fs;M259T**	$2.0x10^{-4}$; $6.5x10^{-3}$	Chet	-	Arabic§	n/a	Portugal
	**p.P84S**	novel	AR	4th	Arabic	MA	USA
	**p.R452***	$1.3x10^{-4}$	AR	3rd	Arabic	MA	Denmark
	**c.607-2A>T**	novel	AR	-	Arabic	MA	NewRad
RNF17, ENST00000255324.5	p.(I796T)	novel	AR	-	EUR	n/a	Portugal
TDRD9 ENST00000339063.5	p.(Y298fs)	novel	AR	4th	Arabic	SCO/MA	USA
	**p.(Q1123*)**	$2.3x10^{-4}$	AR	-	EUR	MA	Spain
	p.(P772fs);(p.Q1123*)	$3.1x10^{-5}$; $2.3x10^{-4}$	Chet	-	EUR	n/a	Portugal
TDRD12 ENST00000421545.2	p.(R109*)	$1.8x10^{-4}$	AR	-	EAS	SCO	Australia
	**p.(N198S)**	$5.1x10^{-4}$	AR	3rd	Arabic	SCO	MERGE
TDRKH ENST00000368827.6	c.1435-2A>G	novel	AR	3rd	EUR	MA	Portugal

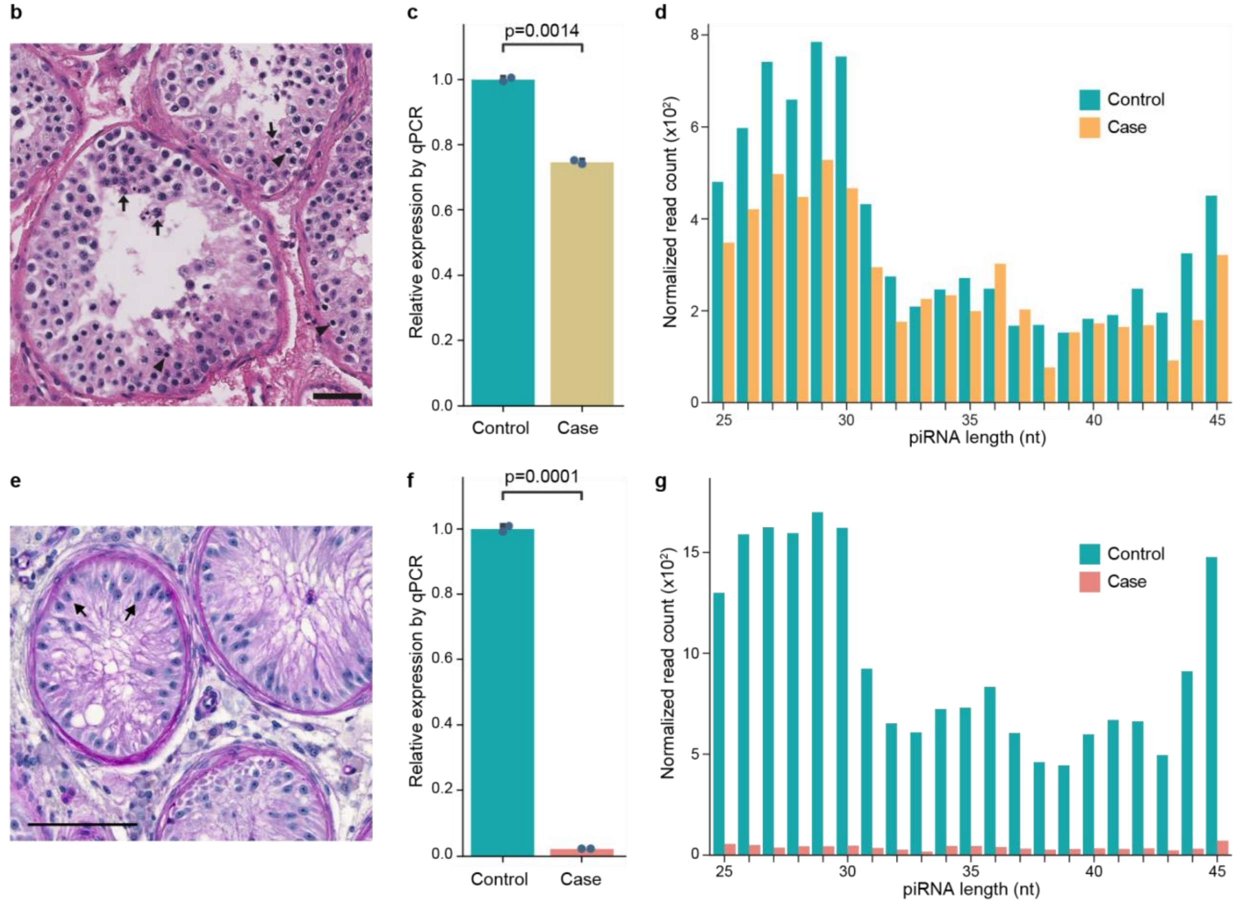

Supplementary Fig. 8. Characteristics and validation of prioritized variation detected in genes relevant for piRNA processing across male infertility cohorts. a, The characteristics of variation in piRNA processing genes. Carriers with testicular tissue available for experimental validation are indicated in bold. *Variant annotation based on ANNOVAR (Gencode v19), †Self-reported descent or inferred ethnicity if no information available, ‡Compound heterozygous variation confirmed to be in trans configuration, §Suggestive Arabic descent based on patient name. AR, autosomal recessive; Chet, compound heterozygous variation; NewRad,

Newcastle/Radboud; SCO, Sertoli Cell Only; MA, maturation arrest; n/a, information unavailable. **b-d**, Validation of a rare stopgain variation p.(Q1123*) in the *TDRD9* gene. **b**, H&E stain of TDRD9 patient biopsy showing spermatogenic arrest. The most mature germ cell observed were early round spermatids, which often appeared multinucleated (arrows). In addition, many pyknotic cells were observed (arrowheads). Scale bar=50 μm. **c**, The RT-qPCR analysis detected significantly reduced levels of the *TDRD9* transcript in the testicular tissue of the p.(Q1123*) carrier compared to the control with normal spermatogenesis. The data are presented as mean values +/- SD from technical duplicates. P values were determined by unpaired two-sided Student's t-test. **d**, Small RNA sequencing of the patient testicular tissue revealed reduced amounts of mature piRNA molecules (<32 nt) in the case compared to the control leading to a significant shift towards longer immature piRNAs (Fig. 5d). **e-g**, Analysis of the testicular tissue from the patient with the missense variation p.(N198S) in the *TDRD12* gene. **e**, The carrier of the p.(N198S) variation displayed Sertoli Cell Only syndrome in all seminiferous tubules, whereby only Sertoli cells are found (arrows) and no germ cells are observed. Testicular tissue was stained with Periodic Acid-Schiff (PAS) and the bar represents 100 µm. **f**, Relative expression of *TDRD12* demonstrated a nearly absent levels of the transcripts in the patient compared to the testicular tissue of a control with normal spermatogenesis. The data are presented as mean values +/- SD from technical duplicates. P values were determined by unpaired two-sided Student's t-test. **g**, Size distribution of piRNAs detected in the NOA case with a biallelic missense variant in *TDRD12* and matching control, derived from small RNA-sequencing of testis tissue.

SUPPLEMENTARY TABLES

Supplementary Table 1. Clinical characteristics of the GEMINI study cohort. None of the measured parameters showed statistically significant differences between the cases with or without a suspected genetic cause. OS, oligozoospermia; Crypt, cryptozoospermia.

		All cases, n=924			Cases with suspected genetic cause, n=184		
Measurement	Range in controls	No. of cases with phenotype info	Mean (range)	SD	No. of cases with phenotype info	Mean (range)	SD
Sperm conc (10^6/ml), NOA	>15	885	0	n/a	177	0	n/a
Sperm conc (10^6/ml), Severe OS/Crypt	>15	9	0.2 (0.01-1.2)	0.41	1	0.01	n/a
s-LH (IU/L)	1.5-6.8	295	8.1 (0-48.8)	5.62	58	7.8 (0.01-24.0)	4.46
s-FSH (IU/L)	1.4-7.6	538	20.4 (0-86.3)	13.53	108	20.2 (0.01-69.8)	13.03
s-Inhibin B (pg/ml)	66.9-300	81	36.9 (1-185)	45.64	12	45.3 (1.0-163.0)	59.27
s-Estradiol (pmol/L)	48-154	73+2*	63.6 (0-139)	25.41	11+1*	65.9 (30.0-117.0)	24.51
s-Total testosterone (nmol/L)	8.7-27.4	300	14.7 (0-88.3)	8.42	58	14.2 (0.1-88.3)	11.63
s-Free testosterone (nmol/L)	4.65-18.1	118+2†	6.0 (0.3-14.2)	2.5	20+1†	5.6 (1.7-10.2)	2.33
Semen volume (ml)	1.8-7.9 ml	89	2.9 (0.2-9.0)	1.54	17	2.9 (0.7-5.0)	1.44
Left testis volume (ml)	34-62 ml	353	11.2 (0-28.0)	5.59	78	11.1 (2-28.0)	5.40
Right testis volume (ml)	34-62 ml	350+1‡	11.7 (0-28.0)	5.91	76	11.7 (0-28.0)	5.72
Age at recruitment (years)	n/a	273	35.5 (16.0-68.7)	7.35	56	35.6 (21.6-50.8)	6.84

*Outliers with s-Estradiol levels 208 and 405 pmol/L excluded from the summary statistics,†Outliers with s-Free testosterone levels 37 and 41 nmol/L excluded from the summary statistics, ‡Outlier presenting with an enlarged right testis (volume 100 ml) was excluded from the summary statistics. The left testis volume of the patient was 13 ml.

Supplementary Table 2. Fraction of individuals with prioritized rare SNVs and INDELs among NOA cases (n=924) and institutional controls (total, n=2265, n=851 men). Note the highest sensitivity of identifying a possible genetic cause of NOA in the autosomal homozygous inheritance mode, which was likely powered by the presence of consanguineous men in the NOA cohort. P-values (Fisher's exact test) below 0.05 were considered significant. *The sum of individuals across disease models is larger than "Total" as multiple candidate disease models can be observed per person.

	NOA cases	Institutional controls			
Disease model	All (count)	All (count)	Fisher's exact P	Men (count)	Fisher's exact P
Single variant autosomal recessive	11.0% (102)	2.0% (46)	7.0×10^{-25}	2.0% (17)	2.4×10^{-15}
Compound-heterozygote autosomal recessive	7.3% (67)	4.0% (91)	2.1×10^{-4}	4.8% (41)	3.7×10^{-2}
X-linked hemizygote	1.7% (16)	0.2% (4)	3.1×10^{-6}	0.5% (4)	1.3×10^{-2}
Y-linked hemizygote	0.4% (4)	0	7.0×10^{-3}	0	0.126
Total*	19.3% (178)	6.0% (135)	1.7×10^{-27}	6.9% (59)	1.0×10^{-14}

Supplementary Table 3. Replication screening of putative NOA disease genes identified in the GEMINI cohort (n=924 cases). The screening was performed in male infertility cohorts from Germany (MERGE, n=817 cases) and UK/Netherlands (Newcastle/Radboud, n=331). Genes altered in all three cohorts are indicated in bold. Azoo, azoospermia; Crypto, cryptozoospermia; HypoSpg, hypospermatogenesis; NewRad, Newcastle/Radboud; NOA, non-obstructive azoospermia; OA, oligoasthenozoospermia

	Number of carriers				Case phenotypes		
Gene name	GEMINI	MERGE	NewRad	Total	GEMINI	MERGE*	NewRad
M1AP	2	4	2	8	2xNOA	3xNOA;Crypto/Oligo	2xNOA
KCTD19	1	1	1	3	NOA	NOA	NOA
YY2	1	1	1	3	NOA	NOA	Severe OA
PNLDC1	3	0	1	4	3xNOA	-	NOA
TGIF2LY	1	2	0	3	NOA	3xNOA	-
VCX	1	3	0	4	NOA	3xNOA	-
BRCA2	1	2	0	3	NOA	2xNOA	-
MROH9	1	0	2	3	NOA	-	2xNOA
NR0B1	1	2	0	3	NOA	NOA;Oligo	-
SPANXN1	1	2	0	3	NOA	2xNOA	-
ZNF543	1	2	0	3	NOA	NOA;Crypto/Oligo	-
AK7	1	1	0	2	NOA	NOA	-
AKAP4	1	1	0	2	NOA	NOA	-
ATP6V0E2	1	1	0	2	NOA	NOA	-
BAX	1	0	1	2	NOA	-	NOA
C9orf84†	1	1	0	2	NOA	Azoo	-
CCDC79	1	1	0	2	NOA	NOA	-
CEP85	1	1	0	2	NOA	Crypto/Oligo	-
CLCN2	1	1	0	2	NOA	NOA	-
DCAF4	1	1	0	2	NOA	NOA	-
DICER1	1	1	0	2	NOA	NOA	-
ESPN	1	1	0	2	NOA	Crypto/Oligo	-
FNDC7	1	1	0	2	NOA	NOA	-
INHBB	1	1	0	2	NOA	NOA	-
MAGEB18	1	1	0	2	NOA	NOA	-
NME7	1	1	0	2	NOA	NOA	-
PMP22	1	0	1	2	NOA	-	Asthenozoospermia
POTEJ	1	1	0	2	NOA	NOA	-
POU4F2	1	1	0	2	NOA	NOA	-
RNF17	1	1	0	2	NOA	NOA	-
SPATA31D1	1	1	0	2	NOA	Crypto/Oligo	-
SPIDR	1	1	0	2	NOA	NOA	-
SPOCD1	1	1	0	2	NOA	NOA	-
STAG3	1	1	0	2	NOA	NOA	-
STPG1	1	1	0	2	NOA	NOA	-
TDRD12	1	1	0	2	NOA	NOA	-
TMEM63C	1	1	0	2	NOA	NOA	-
TUBA3C	1	1	0	2	NOA	Azoo	-
USP9Y	1	1	0	2	NOA	NOA	-
VCX2	1	1	0	2	NOA	NOA	-
ZIC5	1	1	0	2	NOA	NOA	-
ZMYM5	1	1	0	2	NOA	NOA	-

*Alternate phenotypes observed in consecutive semen analyses in a patient are separated by '/'. † Also known as *SHOC1*.

Supplementary Table 4. Burden testing of recurrent GEMINI genes in 2,072 cases and 11,587 fertile parents. A subset of GEMINI genes were selected for burden testing, comparing the frequency of deleterious genotypes in infertility cases against the frequency in proven parents (see Methods for details). We performed two-stage burden testing, using a two-sided Fisher-exact test comparing the the frequency of genotypes in all cases combined (GEMINI+MERGE+NewRad="Case total Count") compared to all controls ("Combined Control Count"). While many p-values are nominally significant, none of the resulting p-values reach exome-wide significance (Methods).

Gene	GEMINI count	Male Control Count	Combined Control Count	MERGE count	NewRad count	Case total count	BurdenTest p-val
AKAP4	1	2	2	1	0	2	0.074
BAX	1	0	1	0	1	2	0.04
BRCA2	1	0	1	1	0	2	0.04
C1orf146	2	0	0	0	0	2	0.015
CCDC89	2	0	0	0	0	2	0.015
CLCN2	1	1	1	1	0	2	0.04
CSMD1	2	1	2	0	0	2	0.074
DCAF12L1	3	0	0	0	0	3	0.002
DCAF4	1	0	0	1	0	2	0.015
FBXO15	3	0	0	0	0	3	0.002
FNDC7	1	0	0	1	0	2	0.015
HCN4	2	0	0	0	0	2	0.015
INHBB	1	0	0	1	0	2	0.015
MAGEB18	1	1	1	1	0	2	0.04
MROH9	1	0	0	0	2	3	0.002
NFKBIB	2	0	0	0	0	2	0.015
NOTCH1	2	1	3	0	0	2	0.113
NR0B1	1	5	5	2	0	3	0.061
PCDHB3	2	1	1	0	0	2	0.04
PMP22	1	1	2	0	1	2	0.074
PNLDC1	2	0	0	0	0	2	0.015
POTEJ	1	0	0	1	0	2	0.015
RNF17	1	1	3	1	0	2	0.113
RNF212	2	0	0	0	0	2	0.015
SLC4A2	2	1	1	0	0	2	0.04
SPANXN1	1	0	0	2	0	3	0.002
SPIDR	1	1	1	1	0	2	0.04
SPOCD1	1	0	0	1	0	2	0.015
TDRD12	1	0	1	1	0	2	0.04
TDRD9	2	0	0	0	0	2	0.015
TGIF2LY	1	1	1	2	0	3	0.006
TMEM63C	1	0	0	1	0	2	0.015
USP9Y	1	3	3	1	0	2	0.113
YY2	1	0	0	1	1	3	0.002

Male control count, count of prioritized genotypes found in just men with proven fertility
Case total count, combined sum of prioritized genotypes in GEMINI+MERGE+NewRad
NewRad, Newcastle/Radboud

Supplementary Table 5. Genes with first instances of human knock-out in human populations based on human variation databases. The genes with 'knock-out' genotypes were screened for similar genotypes in five large human variation databases, including gnomAD, deCODE, Human Gene Mutation Database, but also exome datasets from Born in Bradford, Birmingham project, and East London Genes and Health project.

Case ID	Gene	Amino acid change*	Disease Model	Country	Ethnicity	Testicular phenotype	Overlapping long ROH, Mb (class)†	% autosomes in long ROH	Consanguinity degree
GEMINI-131	ATP6V0E2	p.Arg120Ter	AR	USA	AFR	n/a	11.5 (cl5)	4.8	3
GEMINI-669	AXDND1	p.Arg313Ter	AR	PRT	EUR	n/a	19.5 (cl5)	2.2	n/a
GEMINI-129§	C16orf46	p.Lys6Ter	AR	USA	EUR	MA	21.0 (cl5)	6.8	3
GEMINI-269	CLTC	p.Q1083fs	AR	USA	EUR	MA	12.2 (cl5)	5.3	3
GEMINI-862	DCAF12L1	p.P449fs	XL	ESP	EUR	MA	n/a	1.72	n/a
GEMINI-712	DCAF12L1	p.Gln145Ter	XL	PRT	EUR	MA	n/a	0.4	n/a
GEMINI-464	DCAF12L1	p.Leu136fs	XL	EST	EUR	n/a	n/a	0.2	n/a
GEMINI-716	DCAF4	p.Cys88Ter	AR	PRT	EUR	HSG	8.6	6	3
GEMINI-492	DDX3Y	p.Ser410fs	YL	EST	EUR	n/a	n/a	6.4	n/a
GEMINI-143	FBXO15	p.Ala88fs	AR	USA	EAS	SCO	1.4 (cl3)	9.6	2 or 3
GEMINI-370	HIF3A	p.Trp17Ter	AR	DNK	EUR	MA	n/a	0.2	n/a
GEMINI-930	MAGEA3	p.Gln63Ter	XL	ESP	EUR	MA	n/a	0.6	n/a
GEMINI-158	NEIL2	p.Lys118fs	AR	USA	EUR	n/a	2.3 (cl4)	11.4	2
GEMINI-340	PNLDC1	p.R452Ter	AR	DNK	SAS	MA	34.4 (cl5)	4.9	3
GEMINI-377	C9orf84‡	p.Glu362fs	AR	DNK	EUR	MA	12.2 (cl5)	3.9	4
GEMINI-620	SPIDR	p.Gln143Ter	AR	PRT	EUR	n/a	7.2 (cl5)	3	4
GEMINI-154	ST6GALNAC6	p.Ser8fs	AR	USA	EUR	n/a	2.5 (cl4)	2.2	n/a
GEMINI-101	TGIF2LX	p.Pro189fs	XL	CAN	SAS	n/a	n/a	0.3	n/a
GEMINI-129§	ZFR2	p.Arg781Ter	AR	USA	EUR	MA	2.1 (cl3)	6.8	3
GEMINI-81	ZNF138	p.Arg103Ter	AR	AUS	EUR	n/a	12.7 (cl5)	8.9	2 or 3
GEMINI-315	ZNF782	p.Thr445fs	AR	USA	EUR	SCO	4.6 (cl4)	3.3	4

*Annotations retrieved by ANNOVAR tool (Gencode v19) integrated in the PSAP tool. See Supplementary Table 2 for further variant details.
†ROH regions are stratified by size into 5 classes, the longest of which (class 5) is more likely to be linked to disease and is indicative of recent consanguinity (Methods). ROH regions are not called for sex chromosomes.
‡Also known as SHOC1
§Carrier of two novel gene KOs
AR, single variant autosomal recessive
SCO, Sertoli Cell Only
MA, Maturation Arrest
ROH, Runs of Homozygosity
XL, X-linked
YL, Y-linked

Supplementary Table 6. Known and suspected infertility genes (n=17) disrupted among NOA cases. List of genes linked to male infertility (n=164) was extracted from Oud et al. 2019[23]. Genes with 'limited' evidence should be considered as candidate genes that require additional proof of association. AD, Autosomal dominant, AR, Autosomal recessive, XL, X-linked, MA, Maturation arrest, SCO, Sertoli Cell Only, n/a, information unavailable.

Gene name	Case count	Testicular histology	Disorder	Inheritance pattern	Evidence
TDRD9	3	MA;n/a;n/a	Non-obstructive azoospermia	AR	Limited
NOTCH1	2	SCO; SCO	Isolated hypogonadotropic hypogonadism	AD	Limited
AK7	1	MA	Multiple morphological abnormalities of the sperm flagella	AR	Limited
AKAP4	1	SCO	Multiple morphological abnormalities of the sperm flagella	XL	Limited
CCDC155	1	SCO	Non-obstructive azoospermia	AR	Limited
DMC1	1	n/a	Non-obstructive azoospermia	AR	Limited
NR0B1	1	n/a	Congenital Adrenal Hypoplasia; Isolated hypogonadotrophic hypogonadism or Late-onset adrenal failure; 46,XX Disorders of Sex Development	XL	Definitive; Definitive; Limited
RSPH1	1	n/a	Primary ciliary dyskinesia	AR	Limited
CCDC40	1	SCO	Primary ciliary dyskinesia	AR	Strong
DNMT3B	1	SCO	Non-obstructive azoospermia	AD	Limited
FANCM	1	n/a	Oligozoospermia	AR	Strong
FSIP2	1	SCO	Multiple morphological abnormalities of the sperm flagella	AR	Limited
MAMLD1	1	n/a	46,XY Disorders of Sex Development	XL	Strong
MAP3K1	1	SCO	46,XY Disorders of Sex Development	AD	Limited
PLK4	1	SCO	Azoospermia	AD	Limited
PROK2	1	n/a	Kallmann syndrome	AR	Strong
ZMYND15	1	n/a	Non-obstructive azoospermia	AR	Limited

Supplementary Table 7. Primers used to validate STRA8 deletion and map deletion breakpoints. A homozygous ~6kb deletion on chromosome 7 (chr7:134,925,271-134,931,454, hg19) residing within the *STRA8* gene was identified in an NOA case GEMINI-295 by calling CNVs from WES data. We first validated the presence of this deletion in the patient DNA using +/- PCR with primers anchoring inside the deletion in exons 2-4 as well as outside the deletion in exon 8. Only the second set of primers outside the deletion amplified from the patient DNA as expected (Fig. S6). We then tested four additional pairs of primers in the intronic regions flanking the deletion to further delimit the breakpoint. Finally, we designed primers for amplification across the expected breakpoint (gap-PCR) with a fragment of maximum expected size of 13 kb without the deletion. This primer set only resulted in a single product of ~5 kb in the patient indicative of a homozygous deletion (Supplementary Fig. 5). The acquired PCR product was sequenced and allowed us to determine the precise localization of the deletion breakpoints at chr7:134925172-134933449 (hg19). The 5' breakpoint is nearly equidistant (~400 bp) from an L1ME3 repeat of the L1 family and an MLT1AD repeat of the ERVL-MaLR family.

Forward	Reverse	Amplified in patient DNA?
STRA8_E2_F: AACCCCTGAAGAAAACAGCA	STRA8_E2_R: TTCCTGAGGTTGTTGAAGAGC	No (not shown)
STRA8_E3_E4_F: CTGGAGCAAACCCTGGATAA	STRA8_E3_E4_R: ACCTCCTCTAAGCTGCTTGC	No (PCR1, FigS6)
STRA8_E8_F: CAGCTGCAACCCAGAAAAC	STRA8_E8_R: CTGCTTTACTTGAGTGTTTGCAC	Yes (PCR2, FigS6)
STRA8_E6_F: ACGATGGACCTTCTGACTGG	STRA8_E6_R: CCTCTCCTCCGAGAGGTTCT	No (not shown)
STRA8_I1_F: GGAGGGACACAACTGATGCT	STRA8_I1_R: TCTCTGCCCCTGTCTTCAGT	Yes (not shown)
STRA8_I1.1_F: ATTTCAGGCTGAGCGTGTTT	STRA8_I1.1_R: GGGTGGGAAGCACTGTTCTA	Yes (not shown)
STRA8_I6_F: ACCCTGGGCTCCAATAATCT	STRA8_I6_R: TGAGGGACAGGAAGAAATGG	Yes (not shown)
STRA8_I6.1_F: TCGTGGACATTTAGCATCCA	STRA8_I6.1_R: GGCTGTAACTCCAGGGTCAG	No (not shown)

STRA8_across_F1: CCTGAGACTGTTAGAGAACTTGAGG	STRA8_across_R1: AGGTGTGAGATGCAATTACCAGAG	Yes (PCR3, FigS6)

SUPPLEMENTARY DISCUSSION

Analysis and characteristics of the NOA cohort

The NOA cases were included into the study based on clinical criteria standardized across the GEMINI centers (Methods). We performed WES on 1,011 men with NOA and performed extensive quality control at DNA sample, sequencing and genotyping level. Out of 1,011 men, 14 were excluded due to DNA sample contamination, as detected based on FREEMIX and confirmed by the combination of normalized coverage of chromosome Y and the inbreeding coefficient F of chromosome X (Supplementary Fig. 1a,c), or poor sequencing quality, such as average coverage <30x. We excluded further 30 samples with poor alignment or genotyping metrics and removed one counterpart in four sibling pairs and in six pairs of apparently duplicate or twin cases (Supplementary Fig. 1a).

To ensure a homogeneous collection of cases with unexplained NOA, we used the genotyping and CNV calls of the WES data to confirm the absence of common genetic causes of azoospermia or any severe genomic aberrations that may be responsible for the disease in the cases. We identified 19 men with unknown Klinefelter syndrome and four cases with Y chromosome AZFa/AZFb microdeletions (Supplementary Fig. 1a). These common causes of NOA may have been missed in the patient work-up or the information may not have been available for historical cases. Additionally, we identified unusual large rearrangements on chromosomes X in one man (Supplementary Fig. 1a,c,d) and a number of deletions spanning chromosome Y regions Yp11.31-q11.223 in two and Yp11.2- q11.21 in one man. Two cases were predicted to carry multiple heterozygous autosomal deletions within the region 1p11.2 to q21.1. Translocations involving 1q21 have previously been implicated in non-obstructive azoospermia implying that genes relevant for spermatogenesis are located within the region[2]. As a conservative measure, we also elected to remove five cases who were heterozygous for a single well-acknowledged pathogenic *CFTR* variant as we are not able to exclude the presence of another non-coding pathogenic variant in the *CFTR* gene in trans with a detected coding variant based on the WES data[3,4]. Six cases were found to carry *CFTR* variants listed in the curated collection of pathogenic lesions in the ClinVar database (Expert Panel, July 2018)[5]. One of the cases harbored pathogenic compound heterozygous variants, whereas two cases with singleton *CFTR* variants were simultaneously the carriers of supernumerary X chromosome (46,XXY) or complex chromosome X rearrangements further validating their exclusion from the study. We did not identify any pathogenic variants in the *ADGRG2* gene linked to congenital bilateral absence of vas deferens[6].

Following the quality control measures mentioned above, the final cohort consisted of 924 men with complete azoospermia or in a few instances, very low concentration of spermatozoa in the semen sample (severe oligozoospermia/cryptozoospermia, n=9) for unexplained reasons (jointly termed ‘NOA’) (Supplementary Table 1). The key diagnostic parameters of NOA, apart

from the concentration of spermatozoa in the ejaculate, were at least partially available in 63% of the 924 cases, including testis volume and serum measurements of LH, FSH, total and free testosterone (Supplementary Table 1). Majority of the cases with FSH readings exhibited at least normal or increased levels of the hormone (97% or 84% of cases, respectively) indicative of a loss of negative feedback from the germ cells and consistent with the primary testicular failure (Supplementary Fig. 2). There were no differences in the endocrine, semen or other diagnostic parameters between the cases with or without a molecular cause identified in this study (data not shown). The NOA cohort predominantly included men of European ancestry (91%), followed by South Asia (4%), Africa (3%) and East Asia (2%).

Consanguinity and uniparental isodisomy in NOA cases

Consanguinity

To evaluate the level of consanguinity among the sequenced cases, we identified long runs of homozygosity (ROH) in the sequenced cases based on the WES data. Only the longest class (class 5) of the ROH regions were considered, as these reflect recent consanguinity and are most likely to contribute to disease[7]. The lower boundaries of the class 5 ROH varied by the population from 6.2 Mb in South Asians, 6.7 Mb in Europeans, 6.8 Mb in Africans to 7.2 Mb in East Asians (average across populations 6.7 Mb). The fraction of the autosome in autozygous state (also known as FROH) above 2.6% is indicative of at least 4th degree of consanguinity[8] and was characteristic to 72 men in our study. Out of these men, 22 were predicted to have 4th degree of consanguinity, one bordering on the 4th and 3rd degree, 34 were with 3rd and 7 with either 3rd or 2nd degree. Finally, eight cases were found to have a 2nd degree of consanguinity as a secondary finding in the WES analysis, which is considered reportable according to the ACMG guidelines[9].

The largest fraction of the consanguineous cases was recruited at Weill Cornell Medicine, NY, USA (47.2%, amounting to 16.6% of all cases sequenced per center), which commonly sees patients of Arabic descent, where consanguineous relationships are practiced at a higher rate[10]. In line with a recent report[11], the NOA cases with consanguineous background displayed a tendency towards being diagnosed with maturation arrest compared to non-consanguineous patients (41.0% and 24.5%, respectively, Fisher’s Exact Test P=0.034). As expected, consanguineous men contributed a large fraction (66.4%) of the deleterious single variant autosomal homozygotes detected in this study, majority of which were overlapping long autozygous regions (71.7%; range 6.9-60.5 Mb).

Uniparental isodisomy

On the basis of the ROH data, two unrelated NOA cases from Utah, USA, were identified with uniparental isodisomy (UPD) - one with 97% of chromosome 2 (UPD2) and one with 98% of chromosome 4 (UPD4) in autozygous state (Supplementary Fig. 4a). The prevalence of UPD in

the GEMINI cohort appears higher than recently reported for a general population based on trios from the 23andMe dataset (1:462 vs 1:2000 per birth)(*17*) and significantly so when considering the Utah NOA cases alone (n=62, 1:31, P=4.6x10^{-4}). Although both of the chromosomes are fairly commonly affected by UPD in the general population, an abnormal phenotype is relatively rare and predominantly attributed to pathogenic recessive variants unmasked by isodisomy[12,13]. Only UPD4 has been implicated in male infertility previously (UPD database: http://cs-tl.de/Start.html) as a recessive variant in *GNRHR* was recently linked to normosmic congenital hypogonadotropic hypogonadism[14]. The UPD4 carrier identified in this study was diagnosed with SCO and Type I diabetes in addition to NOA. A single potentially deleterious variant was prioritized in the case, a nonsense change p.(Arg761*) (ENST00000382865.1:exon20:c.C2281T; MAF=2x10^{-5}; rs762214513), predicted to prompt nonsense-mediated decay, in the *POLN* gene located on the isodisomic chromosome 4. *POLN* mediates DNA repair and homologous recombination[15] and although disruption of *Poln* in mouse yielded normal reproductive phenotype, certain meiotic recombination defects were observed in the testis[16]. The UPD2 case and the underlying *INHBB* variation p.(Met360Thr) (ENST00000295228.3:exon2:c.T1079C; MAF=2x10^{-5}, rs746066693) have previously been reported in the GEMINI pilot study by Lopes et al. 2013[17]. *INHBB* encodes the beta subunit of inhibin B known to suppress pituitary FSH production and its circulating levels are sometimes used as a clinical readout of spermatogenic output[18]. Extended phenotype information was unavailable for this case. Neither of the variants were overlapping CNVs confirming the autozygosity of the respective regions.

Description of copy number variation

Rare copy number variants detected in the WES data (<1% among the NOA cases) were subjected to PSAP analysis and downstream prioritization jointly with the detected SNVs and INDELs (Supplementary Fig. 3). As a result, a total of five CNVs were prioritized in six men (Supplementary Fig. 5a). These included one 1.8 kb duplication of two exons of an X-linked gene *CT47B1,* a cancer-testis antigen family member, and five large deletions (0.48 kb-1.1 Mb) that affected another five genes. All of the genes found overlapping the CNV regions were novel in the context of infertility in men, except for *STRA8*, which has an essential role in spermatogenesis as a meiotic gatekeeper and was found disrupted in an NOA cohort[19,20]. The largest predicted hemizygous deletion of 1.1 Mb on chromosome X was observed in a Portuguese case partially or fully deleting five genes - *PUDP, STS, PNPLA4, VCX* and *VCX2*. The latter two were prioritized by the analysis pipeline as the most likely disease gene candidates and represent another duplicate cancer-testis antigen family with an exclusive expression in testicular germ cells and cancer but poorly understood function in spermatogenesis[21,22]. The large deletion was tested by +/- PCR targeting the coding regions of three genes within the predicted CNV region and flanking the *VCX* gene (exon 1 of *PUDP*, exon 1 of *STS* and exon 7 of *PNPLA4*). None of the targeted regions were amplified indicating the loss of the tested genes but also a full deletion of *VCX*, whereas the PCR targeting exone 10 of the *FAMA9* gene outside the predicted deletion, was positive. The 1.1 Mb deletion was

predicted to remove only the last exon of *VCX2* (exon 3), which was not evaluated experimentally due to high sequence similarity to *VCX*.

A recurrent deletion removing the whole genic region of the *U2AF1* gene was identified in two unrelated NOA cases from different centers, Australia and USA (Supplementary Fig. 5a). Although the role of spliceosome component U2AF1 in germ cell development is unclear, the knockout of its paralog *Zrsr1* leads to azoospermia in mice due to increased intron retention in genes relevant for spermatogenesis[23]. A 476 bp deletion was identified within the *HBA2* gene encoding a hemoglobin alpha chain, which is exclusively detected in the whole blood (GTEx database) and likely represents a false positive association to NOA. The HBA2 as well as the U2AF1 deletions were confirmed by +/- PCR.

<u>Overview of NOA cases with variants in known syndromic male infertility genes</u>

We noted that a subset of our prioritized NOA genes have previously been linked to other forms of male infertility, indicating that variants in some testis-expressed genes could have variable presentation depending on the nature of the particular DNA change, the genetic background of the individual, environmental exposures and perhaps, chance. The genes affected have previously been linked to isolated hypogonadotropic hypogonadism, Kallmann Syndrome, multiple morphological abnormalities of the sperm flagella (MMAF) and primary ciliary dyskinesia (PCD) (Extended Data Table 2). Here we describe in greater detail these specific cases and the implications.

We have identified recessive variation within the genes *PROK2* and *NOTCH1* that have previously been linked to Kallmann syndrome and Isolated hypogonadotropic hypogonadism respectively[24]. Constitutively active NOTCH1 has been shown to cause depletion of gonocytes in mice[25], whereas *PROK2*, which is expressed in undifferentiated spermatogonia and spermatocytes ('HISTA' scRNAseq visualization tool at https://conradlab.shinyapps.io/HISTA)[26], has been proposed as a regulator of testicular inflammation[27].

Three NOA cases were identified with potentially deleterious variants in *PROK2* or *NOTCH1*, without displaying the classical diagnostic symptoms of the Kalmann syndrome and isolated hypogonadotropic hypogonadism (Extended Data Table 2). A pair of putatively compound heterozygous missense variants in the *PROK2* gene (ENST00000353065.3:exon3:c.C250T:p.His84Tyr, rs201632855 and ENST00000353065.3:exon2:c.G122A:p.Gly41Asp, rs200922174) was identified in a man diagnosed with NOA and with no evidence of syndromic infertility. The man displayed high FSH 25.1 IU/L and LH 9.38 IU/L as commonly observed in isolated NOA patients, testosterone 16.2 nmol/L, bioavailable testosterone 8.4 nmol/L and the volume of both testes of 16 ml. No sperm was recovered in testicular sperm extraction (TESE) and the olfactory phenotype linked to Kallmann syndrome was not present. The frequency of these variants across gnomAD exome data remains low (max MAF=0.0001 for p.His84Tyr and 0.0013 for p.Gly41Asp) and both variants are predicted to be deleterious based on the CADD scores (31 and 28.9, respectively)

and multiple prediction algorithms (disease causing by MutationTaster, probably damaging by Polyphen and deleterious by SIFT for both variants).

Similarly, two patients with homozygous missense variants in *NOTCH1* were found in two men diagnosed with SCO. A carrier of ENST00000277541.6:exon7:c.C1250T:p.Ser417Leu (maximum gnomAD MAF=6.7x10^{-5}, CADD score 32, rs757631575) had FSH levels at 35.2 IU/L, testicular volume of left testis 14 ml and right testis 8 ml and no sperm recovered at TESE. The other patient was a carrier of ENST00000277541.6:exon34:c.C6227T:p.Thr2076Ile (CADD score 28.2, rs1022510242), which has been observed in a single heterozygous non-Finnish European man from gnomAD (MAF=3x10^{-5}). No further phenotype information was available for this patient. Both variants were uniformly predicted to be deleterious (MutationTaster, Polyphen and SIFT). The homozygous state of these rare variants appears to be caused by consanguinity, which is characteristic to both men. The p.Ser417Leu carrier displayed 2.6% and p.Thr2076Ile carrier 5.0% of autosomes in long ROH regions indicative of 4th and 3rd degree of consanguinity, respectively.

We also identified genotypes potentially causal for NOA in six genes linked to ciliary disorders (*AKAP4*, *AK7*, *FSIP2, NEK10*, *CCDC40* and *RSPH1;* Extended Data Table 2)[24,28]. This observation suggests that defects in proteins used during spermiogenesis can present as a spectrum of phenotypes, consistent with the observation of severe oligozoospermia in some patients with MMAF[29,3031]. High variability in genotype-phenotype mapping of genes involved in male gonad function has previously been observed for various genes, including *DMRT1*, where deleterious variants present a spectrum of traits from complete XY sex-reversal to testicular cancer and isolated non-obstructive azoospermia[17,32–34].

Experimental validation of deleterious variation identified in genes regulating piRNA biogenesis

We set out to experimentally validate the functional consequences of prioritized variants found in piRNA processing genes (Supplementary Fig. 8a). Testicular tissue was available for six patients with potentially pathogenic variation in *PNLDC1* (four men from Portugal, USA, Denmark and Newcastle/Radboud cohort from Netherlands) and singleton patients with rare variants in *TDRD9* (Spain) and *TDRD12* (MERGE cohort from Germany). All the detected variants in the three piRNA processing genes have been confirmed by Sanger sequencing. The disruptions of piRNA trimmer *PNLDC1* induced (i) azoospermia with predominantly maturation arrest findings at the pachytene period, (ii) significantly reduced PNLDC1 mRNA and protein levels in testis, (iii) down-regulation of other piRNA processing enzymes, like PIWIL1 and MYBL, and (iv) altered distribution of piRNA lengths detected from small RNA sequencing of the testicular tissue[35]. These findings are largely consistent with the observations in male mice deficient for *Pnldc1*[36–38].

Similar to *PNLDC1*, the variants identified in *TDRD9* and *TDRD12* were extremely rare or not observed in the gnomAD human variation database previously (Supplementary Fig. 8a). Three different biallelic loss-of-function variants were identified in the *TDRD9* gene, which is

considered essential for silencing Line-1 retrotransposons and maintaining DNA stability in the male germ line and causes maturation arrest of germ cells upon disruption in mice[39]. Testicular tissue was available for the carrier of the stopgain p.(Q1123*), which is found at a popmax minor allele frequency of $2.3x10^{-4}$, is located in exon 34 out of 36 total and may potentially trigger nonsense-mediated decay of the affected transcripts. The p.(Q1123*) carrier was a non-consanguienous NOA patient recruited in Spain, had 46,XY karyotype, FSH 4.01 UI/L, LH 3.18 IU/L, total testosterone 8.7 nmol/L, testes volumes of 20 ml and no history of testicular trauma, orchitis, cryptorchidism, infection or varicocele. The patient displayed incomplete spermatocyte arrest with a few spermatids observed in some tubules (Supplementary Fig. 8b) but no spermatozoa recovered by TESE. This is consistent with a previously reported frameshift deletion in *TDRD9,* which was identified in a consanguineous Bedouin family affected by NOA and led to incomplete maturation arrest among the carriers[40]. In the patient with p.(Q1123*), the levels of *TDRD9* transcripts were significantly reduced in testicular tissue when compared to controls with complete spermatogenesis (Supplementary Fig. 8c). We were unable to get a commercial antibody directed against TDRD9 to work properly and hence could not verify TDRD9 protein levels in the patient. However, small RNA sequencing revealed a reduced fraction of mature piRNA molecules (<32 bases) and accumulation of larger immature piRNAs (>32 bases) (Fig. 4d, Supplementary Fig. 8d).

In *TDRD12*, two rare homozygous variants, a stopgain p.(R109*) and a missense variation p.(N198S), were detected in NOA cases from Australia and Germany (MERGE cohort), respectively (Supplementary Fig. 8a). Like *TDRD9, TDRD12* is relevant for retrotransposon repression and secondary biogenesis of piRNAs and causes male-specific infertility when disrupted not only in mice, but also zebrafish[41,42]. Variation in *TDRD12* have previously not been linked to infertility phenotypes in men. The testicular tissue was available for the carrier of p.(N198S), which is found in the gnomAD database with a popmax minor allele frequency of $5.1x10^{-4}$ and no homozygous genotypes. The patient with p.(N198S) was recruited in Germany at the age of 26 after trying to conceive for 1.5 years. He originated from Morocco and was a consanguineous descendant of parents who are first degree cousins. He had azoospermia, testicular volumes of 7 ml for both testes, FSH 11.2 U/L, LH 3.1 U/l, testosterone 25.5 nmol/L, 46,XY karyotype and did not have AZF deletions. No germ cells were retrieved by TESE and the testicular biopsy revealed complete Sertoli Cell Only syndrome in all seminiferous tubules (Supplementary Fig. 8e), a more severe phenotype as compared to the $Tdrd12^{-/-}$ male mice, who display maturation arrest at zygotene-pachytene transition and increased levels of germ cell apoptosis (*41*). In accordance with the histological findings, the testicular *TDRD12* transcript levels were significantly reduced (Supplementary Fig. 8f) and the sequencing of small RNAs of the testicular tissue revealed a dramatic loss in piRNAs with fewer mature (<32 nt) piRNAs detected in the case (Fig. 4d, Supplementary Fig. 8g). Collectively, these results are indicative of a collapse of the piRNA biogenesis pathway consistent with the patient's phenotype but also with the observations made in the model organisms deficient for *Tdrd12*[41,42].

These findings add to the observations made for the *PNLDC1* variation carriers[35] and emphasize the critical role of the components of the piRNA biogenesis pathway in maintaining germ cell development and fertility in men.

SUPPLEMENTARY REFERENCES

1. GTEx Consortium. The GTEx Consortium atlas of genetic regulatory effects across human tissues. *Science* **369**, 1318–1330 (2020).
2. Li, R. *et al.* Chromosome 1q21 translocation and spermatogenesis failure: Two case reports and review of the literature. *Medicine* **98**, e18588 (2019).
3. Ge, B. *et al.* A rare frameshift variant in trans with the IVS9-5T allele of CFTR in a Chinese pedigree with congenital aplasia of vas deferens. *Journal of Assisted Reproduction and Genetics* vol. 36 2541–2545 Preprint at https://doi.org/10.1007/s10815-019-01617-4 (2019).
4. de Souza, D. A. S., Faucz, F. R., Pereira-Ferrari, L., Sotomaior, V. S. & Raskin, S. Congenital bilateral absence of the vas deferens as an atypical form of cystic fibrosis: reproductive implications and genetic counseling. *Andrology* **6**, 127–135 (2018).
5. Landrum, M. J. *et al.* ClinVar: improving access to variant interpretations and supporting evidence. *Nucleic Acids Res.* **46**, D1062–D1067 (2018).
6. Patat, O. *et al.* Truncating Mutations in the Adhesion G Protein-Coupled Receptor G2 Gene ADGRG2 Cause an X-Linked Congenital Bilateral Absence of Vas Deferens. *Am. J. Hum. Genet.* **99**, 437–442 (2016).
7. Pemberton, T. J. & Szpiech, Z. A. Relationship between Deleterious Variation, Genomic Autozygosity, and Disease Risk: Insights from The 1000 Genomes Project. *Am. J. Hum. Genet.* **102**, 658–675 (2018).
8. Sund, K. L. *et al.* Regions of homozygosity identified by SNP microarray analysis aid in the diagnosis of autosomal recessive disease and incidentally detect parental blood relationships. *Genetics in Medicine* vol. 15 70–78 Preprint at https://doi.org/10.1038/gim.2012.94 (2013).
9. Rehder, C. W. *et al.* American College of Medical Genetics and Genomics: standards and

guidelines for documenting suspected consanguinity as an incidental finding of genomic testing. *Genet. Med.* **15**, 150–152 (2013).

10. Tadmouri, G. O. *et al.* Consanguinity and reproductive health among Arabs. *Reprod. Health* **6**, 17 (2009).
11. Özman, O. & Bakircioğlu, M. E. The Clinical Impact of Parental Consanguineous Marriage in Idiopathic Non-obstructive Azoospermia. *F&S Reports* Preprint at https://doi.org/10.1016/j.xfre.2020.07.002 (2020).
12. Shaffer, L. G. *et al.* American College of Medical Genetics statement of diagnostic testing for uniparental disomy. *Genet. Med.* **3**, 206–211 (2001).
13. Nakka, P. *et al.* Characterization of Prevalence and Health Consequences of Uniparental Disomy in Four Million Individuals from the General Population. *Am. J. Hum. Genet.* **105**, 921–932 (2019).
14. Cioppi, F. *et al.* Genetics of ncHH: from a peculiar inheritance of a novel GNRHR mutation to a comprehensive review of the literature. *Andrology* **7**, 88–101 (2019).
15. Moldovan, G.-L. *et al.* DNA polymerase POLN participates in cross-link repair and homologous recombination. *Mol. Cell. Biol.* **30**, 1088–1096 (2010).
16. Takata, K.-I. *et al.* Analysis of DNA polymerase ν function in meiotic recombination, immunoglobulin class-switching, and DNA damage tolerance. *PLoS Genet.* **13**, e1006818 (2017).
17. Lopes, A. M. *et al.* Human spermatogenic failure purges deleterious mutation load from the autosomes and both sex chromosomes, including the gene DMRT1. *PLoS Genet.* **9**, e1003349 (2013).
18. Kumanov, P., Nandipati, K., Tomova, A. & Agarwal, A. Inhibin B is a better marker of spermatogenesis than other hormones in the evaluation of male factor infertility. *Fertil. Steril.* **86**, 332–338 (2006).
19. Anderson, E. L. *et al.* Stra8 and its inducer, retinoic acid, regulate meiotic initiation in both

spermatogenesis and oogenesis in mice. *Proc. Natl. Acad. Sci. U. S. A.* **105**, 14976–14980 (2008).

20. Alhathal, N. *et al.* A genomics approach to male infertility. *Genet. Med.* **22**, 1967–1975 (2020).
21. Zou, S. W. *et al.* Expression and localization of VCX/Y proteins and their possible involvement in regulation of ribosome assembly during spermatogenesis. *Cell Res.* **13**, 171–177 (2003).
22. Lahn, B. T. & Page, D. C. A human sex-chromosomal gene family expressed in male germ cells and encoding variably charged proteins. *Hum. Mol. Genet.* **9**, 311–319 (2000).
23. Horiuchi, K. *et al.* Impaired Spermatogenesis, Muscle, and Erythrocyte Function in U12 Intron Splicing-Defective Zrsr1 Mutant Mice. *Cell Reports* vol. 23 143–155 Preprint at https://doi.org/10.1016/j.celrep.2018.03.028 (2018).
24. Oud, M. S. *et al.* A systematic review and standardized clinical validity assessment of male infertility genes. *Hum. Reprod.* **34**, 932–941 (2019).
25. Garcia, T. X., DeFalco, T., Capel, B. & Hofmann, M.-C. Constitutive activation of NOTCH1 signaling in Sertoli cells causes gonocyte exit from quiescence. *Dev. Biol.* **377**, 188–201 (2013).
26. Mahyari, E. *et al.* Comparative single-cell analysis of biopsies clarifies pathogenic mechanisms in Klinefelter syndrome. *Am. J. Hum. Genet.* **108**, 1924–1945 (2021).
27. Li, Y. *et al.* Effects of prokineticin 2 on testicular inflammation in rats. *Am. J. Reprod. Immunol.* **79**, e12843 (2018).
28. Chivukula, R. R. *et al.* A human ciliopathy reveals essential functions for NEK10 in airway mucociliary clearance. *Nat. Med.* **26**, 244–251 (2020).
29. Dong, F. N. *et al.* Absence of CFAP69 Causes Male Infertility due to Multiple Morphological Abnormalities of the Flagella in Human and Mouse. *Am. J. Hum. Genet.* **102**, 636–648 (2018).

30. Tang, S. *et al.* Biallelic Mutations in CFAP43 and CFAP44 Cause Male Infertility with Multiple Morphological Abnormalities of the Sperm Flagella. *Am. J. Hum. Genet.* **100**, 854–864 (2017).

31. Martinez, G. *et al.* Whole-exome sequencing identifies mutations in FSIP2 as a recurrent cause of multiple morphological abnormalities of the sperm flagella. *Hum. Reprod.* **33**, 1973–1984 (2018).

32. Raymond, C. S. *et al.* A region of human chromosome 9p required for testis development contains two genes related to known sexual regulators. *Hum. Mol. Genet.* **8**, 989–996 (1999).

33. Ledig, S., Hiort, O., Wünsch, L. & Wieacker, P. Partial deletion of DMRT1 causes 46,XY ovotesticular disorder of sexual development. *Eur. J. Endocrinol.* **167**, 119–124 (2012).

34. Turnbull, C. *et al.* Variants near DMRT1, TERT and ATF7IP are associated with testicular germ cell cancer. *Nat. Genet.* **42**, 604–607 (2010).

35. Nagirnaja, L. *et al.* Variant , Defective piRNA Processing, and Azoospermia. *N. Engl. J. Med.* **385**, 707–719 (2021).

36. Nishimura, T. *et al.* PNLDC1, mouse pre-piRNA Trimmer, is required for meiotic and post-meiotic male germ cell development. *EMBO Rep.* **19**, (2018).

37. Ding, D. *et al.* PNLDC1 is essential for piRNA 3' end trimming and transposon silencing during spermatogenesis in mice. *Nat. Commun.* **8**, 819 (2017).

38. Zhang, Y. *et al.* An essential role for PNLDC1 in piRNA 3' end trimming and male fertility in mice. *Cell Res.* **27**, 1392–1396 (2017).

39. Shoji, M. *et al.* The TDRD9-MIWI2 complex is essential for piRNA-mediated retrotransposon silencing in the mouse male germline. *Dev. Cell* **17**, 775–787 (2009).

40. Arafat, M. *et al.* Mutation in TDRD9 causes non-obstructive azoospermia in infertile men. *J. Med. Genet.* **54**, 633–639 (2017).

41. Pandey, R. R. *et al.* Tudor domain containing 12 (TDRD12) is essential for secondary PIWI

interacting RNA biogenesis in mice. *Proc. Natl. Acad. Sci. U. S. A.* **110**, 16492–16497 (2013).

42. Dai, X. *et al.* Tdrd12 Is Essential for Germ Cell Development and Maintenance in Zebrafish. *Int. J. Mol. Sci.* **18**, (2017).
