# Supplementary material for: Diverse monogenic subforms of human spermatogenic failure
Source: Nat Commun. 2022 Dec 26;13:7953. doi: 10.1038/s41467-022-35661-z (PMC9792524; doi:10.1038/s41467-022-35661-z)
Supplement: Supplementary file 2 — Description of Additional Supplementary Files [file 41467_2022_35661_MOESM2_ESM.pdf]

DESCRIPTION OF ADDITIONAL SUPPLEMENTARY FILES

For: Diverse Monogenic Subforms of Human Spermatogenic Failure

File: Supplementary Data 1

Description: Prioritized variation identified in NOA cases.

File: Supplementary Data 2

Description: Clinical variant interpretation of GEMINI cases using ACMG guidelines.

File: Supplementary Data 3

Description: Recurrently disrupted genes in NOA cases in the GEMINI cohort.

File: Supplementary Data 4.

Description: Prioritized NOA genes linked to Mendelian diseases in OMIM database.

File: Supplementary Data 5

Description: List of genes linked to various male infertility disorders and plotted in Fig. 3c
