# Supplementary material for: Diverse monogenic subforms of human spermatogenic failure
Source: Nat Commun. 2022 Dec 26;13:7953. doi: 10.1038/s41467-022-35661-z (PMC9792524; doi:10.1038/s41467-022-35661-z)
Supplement: Supplementary file 8 — Reporting Summary [file 41467_2022_35661_MOESM8_ESM.pdf]

nature portfolio

Corresponding author(s): Don Conrad

Last updated by author(s): Nov 23, 2022

Reporting Summary

Nature Portfolio wishes to improve the reproducibility of the work that we publish. This form provides structure for consistency and transparency in reporting. For further information on Nature Portfolio policies, see our Editorial Policies and the Editorial Policy Checklist.

Statistics

For all statistical analyses, confirm that the following items are present in the figure legend, table legend, main text, or Methods section.

n/a	Confirmed	
☐	☒	The exact sample size (n) for each experimental group/condition, given as a discrete number and unit of measurement
☐	☒	A statement on whether measurements were taken from distinct samples or whether the same sample was measured repeatedly
☐	☒	The statistical test(s) used AND whether they are one- or two-sided<br>*Only common tests should be described solely by name; describe more complex techniques in the Methods section.*
☒	☐	A description of all covariates tested
☒	☐	A description of any assumptions or corrections, such as tests of normality and adjustment for multiple comparisons
☐	☒	A full description of the statistical parameters including central tendency (e.g. means) or other basic estimates (e.g. regression coefficient) AND variation (e.g. standard deviation) or associated estimates of uncertainty (e.g. confidence intervals)
☐	☒	For null hypothesis testing, the test statistic (e.g. F, t, r) with confidence intervals, effect sizes, degrees of freedom and P value noted<br>*Give P values as exact values whenever suitable.*
☒	☐	For Bayesian analysis, information on the choice of priors and Markov chain Monte Carlo settings
☒	☐	For hierarchical and complex designs, identification of the appropriate level for tests and full reporting of outcomes
☐	☒	Estimates of effect sizes (e.g. Cohen's d, Pearson's r), indicating how they were calculated

Our web collection on statistics for biologists contains articles on many of the points above.

Software and code

Policy information about availability of computer code

Data collection	bwa-mem v0.7.17, Genome Analysis Toolkit v3.6.0. (GATK), Picard tools v2.10.0., XHMM, bowtie v1.0.1
Data analysis	PSAP (https://github.com/conradlab/PSAP/), pHASER v1.1.1, VerifyBamID v1.1., mclust R package v.5.4.3, IGV tools, EthSeq v2.1.4, H3M2, SNPRelate v1.32.0, LOFTEE v1.0.3., Variant Effect Predictor v99, Cytoscape platform v3.7.2, clusterProfiler R package, SIFT, PolyPhen-2, MutationTaster, FATHMM v2.3, Mutation Assessor, CADD v1.6

For manuscripts utilizing custom algorithms or software that are central to the research but not yet described in published literature, software must be made available to editors and reviewers. We strongly encourage code deposition in a community repository (e.g. GitHub). See the Nature Portfolio guidelines for submitting code & software for further information.

Data

Policy information about availability of data

All manuscripts must include a data availability statement. This statement should provide the following information, where applicable:

- Accession codes, unique identifiers, or web links for publicly available datasets
- A description of any restrictions on data availability
- For clinical datasets or third party data, please ensure that the statement adheres to our policy

- scRNA-seq of testicular cells from a total of 12 human donors, including 7 adults (GEO accession #GSE109037), and two juvenile samples (GEO accession (#GSE120506).
- Sequencing data that support the findings of this study are in process of being deposited in dbGAP database

Field-specific reporting

Please select the one below that is the best fit for your research. If you are not sure, read the appropriate sections before making your selection.

☒ Life sciences ☐ Behavioural & social sciences ☐ Ecological, evolutionary & environmental sciences

For a reference copy of the document with all sections, see nature.com/documents/nr-reporting-summary-flat.pdf

Life sciences study design

All studies must disclose on these points even when the disclosure is negative.

Sample size	We sequenced all cases available through the GEMINI consortium centers, and combined these cases with two other cohorts. To our knowledge this represents the largest collection of unrelated NOA cases to date and is sufficient for exploring rare recessive Mendelian mutations that are predominantly expected to be case-specific.
Data exclusions	The sequencing data underwent extensive filtering to acquire a high-quality genotype call set and the analysis focused exclusively on the identification of recessive genetic causes which is the main cause of male infertility based on the current understand of the disease genetics. All the filtering and exclusion steps are provided in the manuscript, as well as the patient exclusion criteria. As our study aimed to understand the genetic causes of male infertility, we only admitted subjects that were clinically identified as male into the cases cohorts. The control cohorts consisted of both males and females, and relied upon self-reported sex. The source data provided in Supplementary table 4 allows the reader to identify sex-specific frequency of deleterious genotypes in our genes of interest in control cohorts. Our analysis of published mouse fertility phenotypes only considers the fertility status of male mice, as detailed in the manuscript.
Replication	Two additional cohorts of unrelated infertile men were incorporated for screening deleterious variants within genes that were linked to NOA based on the GEMINI study samples. The results confirmed the findings and highlighted a subset of genes recurrently affected across at least two cohorts.
Randomization	We employed a variant prioritization tool Population Sampling Probability (PSAP), which enables the identification of potential causal variants from a single genome without the need for matching control samples. PSAP evaluates the probability of sampling a genotype or a set of genotypes based on the pathogenicity scores and frequencies of variants observed in the unaffected population. Randomization was thus not relevant to our study.
Blinding	As this study did not allocate samples into different patient/control groups, the blinding was not relevant to our study.

Reporting for specific materials, systems and methods

We require information from authors about some types of materials, experimental systems and methods used in many studies. Here, indicate whether each material, system or method listed is relevant to your study. If you are not sure if a list item applies to your research, read the appropriate section before selecting a response.

Materials & experimental systems

n/a	Involved in the study	
☐	☒	Antibodies
☒	☐	Eukaryotic cell lines
☒	☐	Palaeontology and archaeology
☒	☐	Animals and other organisms
☐	☒	Human research participants
☒	☐	Clinical data
☒	☐	Dual use research of concern

Methods

n/a	Involved in the study	
☒	☐	ChIP-seq
☒	☐	Flow cytometry
☒	☐	MRI-based neuroimaging

Antibodies

Antibodies used	Stra8 antibody: Abcam, Cambridge, UK; Cat nr. ab49405. The other antibodies used and optimizations performed are provided in detail in Nagirnaja et al. 2021, NEJM.
Validation	STRA8 was validated for immunohistochemistry and Western blot applications by the manufacturer.

Human research participants

Policy information about studies involving human research participants

Population characteristics	The study participants represent men diagnosed with non-obstructive azoospermia (and cryptozoospermia in a few cases) aged between 16 and 69 years at the time of recruitment. These men underwent assessment of reproductive issues and

collection of information on other past or current afflictions unrelated to fertility was not in the scope of this study. The same applies to treatments, with an exception of any procedures or factors that may directly influence the reproductive outcome, such as radical pelvic surgery, spinal cord injury, radiation treatments or chemotherapy exposure and environmental factors. This data is routinely collected during patient workup with fertility issues aiming to delineate the underlying cause of the reproductive disease. Cases with chromosomal aneuploidies or Y-chromosome deletions were excluded from the study according to the information provided by the recruitment centers or detected based on the sequencing data generated in this study.

Recruitment	Study participants represent retrospective or prospective cases with non-obstructive azoospermia/cryptozoospermia as identified by trained fertility specialists at each recruitment center based on a variety of clinical parameters and according to well-established clinical guidelines.
Ethics oversight	Each collaborative center in the GEMINI consortium performed the patient recruitment under local ethics oversight of respective centers. As also given in the chapter "Ethics statements & IRBs from centers", this included institutional review board (IRB) of Washington University in St. Louis, USA; IRB of University of Utah, USA; Pthe Ethics Committee and Hospital Authority, University of Porto, Portugal; the IRB of Weill Cornell Medical College, New York, USA; the IRB of Fundació Puigvert, Barcelona, Spain; the Research Ethics Committee of the Radboud University, Nijmegen, The Netherlands; the University Research Ethics Committee of University of Newcastle, UK; and ethical approvals from human ethics committees of Monash Surgical Day Hospital, Monash Medical Centre and Monash University, Australia; the ethics committee for the Capital Copenhagen Region and the Danish Personal Data Protection Agency; the Ethics Committee of National Institute of Health Dr Ricardo Jorge, Lisboa, Portugal; Research Ethics Committee of the University of Tartu, Estonia. The MERGE study protocol was given ethical approval by the Ethics Committee of the Ärztekammer Westfalen-Lippe and the University of Münster.

Note that full information on the approval of the study protocol must also be provided in the manuscript.
